# Do HIV-1 non-B subtypes differentially impact resistance mutations and clinical disease progression in treated populations? Evidence from a systematic review

**DOI:** 10.7448/IAS.17.1.18944

**Published:** 2014-07-04

**Authors:** Madhavi Bhargava, Jorge Martinez Cajas, Mark A Wainberg, Marina B Klein, Nitika Pant Pai

**Affiliations:** 1 Division of Clinical Epidemiology & Infectious Diseases, McGill University Health Centre, Montreal, Canada; 2 Division of Infectious Diseases, Department of Medicine, Queen’s University, Kingston, Canada; 3 Lady Davis Institute, Jewish General Hospital, McGill University, Montreal, Canada; 4 Chronic Viral Illness Service, Division of Infectious Diseases & Clinical Epidemiology, Department of Medicine, McGill University and McGill University Health Centre, Montreal, Canada; 5 Department of Medicine, McGill University, Montreal, Canada

**Keywords:** HIV-1, non-B subtypes, disease progression, resistance mutation, differential impact, systematic review, evidence

## Abstract

There are 31 million adults living with HIV-1 non-B subtypes globally, and about 10 million are on antiretroviral therapy (ART). Global evidence to guide clinical practice on ART response in HIV-1 non-B subtypes remains limited. We systematically searched 11 databases for the period 1996 to 2013 for evidence. Outcomes documented included time to development of AIDS and/or death, resistance mutations, opportunistic infections, and changes in CD4 cell counts and viral load. A lack of consistent reporting of all clinical end points precluded a meta-analysis. In sum, genetic diversity that precipitated differences in disease progression in ART-naïve populations was minimized in ART-experienced populations, although variability in resistance mutations persisted across non-B subtypes. To improve the quality of patient care in global settings, recording HIV genotypes at baseline and at virologic failure with targeted non-B subtype-based point-of-care resistance assays and timely phasing out of resistance-inducing ART regimens is recommended.

## Introduction

We are in the fourth decade of the HIV pandemic with 34 million people living with HIV. Of the 26 million eligible for antiretroviral treatment (ART), only 9.7 million are currently on ART, constituting about 37% of the total population in need of therapy [1]. With a rapid ART expansion in global settings, coupled with widespread prevalence of HIV-1 non-B subtypes, there is a great need to understand clinical outcomes in HIV-1 non-B subtypes.

HIV features high replication and mutagenesis rate that generates several subtypes. Variants of the type 1 virus (HIV-1) are grouped into three major phylogenetic groups (M, O and N) [2]. The majority of the infections worldwide are caused by M group, which contains 10 subtypes (A to K) with different prevalence and geographical distribution. In Africa and Asia, 90% of individuals living with HIV have HIV-1 non-B subtypes [3]. Of these, subtype C accounts for more than 50% [2,4] and is mostly present in sub-Saharan Africa, India and Brazil. Subtype A, the second most common, is found in eastern Europe and northern Asia followed by subtype CRF01_AE in southeast Asia and CRF02_AG in West Africa [5]. Many other non-B subtypes and circulating recombinant forms (CRFs) are being reported with increased travel and immigration [6–9]. Despite the known genetic diversity of HIV and the potential emergence of new variants [10,11], the impact of subtype diversity on initiation and sequencing of ART regimens, resistance mutations and clinical disease progression remains relatively unknown.

In 2008, a narrative review synthesized the presence of differences in subtype transmission, co-receptor usage, disease progression and response to ARTs amongst non-B subtypes [12]. Subsequently, studies alluded to evidence of no difference in HIV disease progression between naive and treated B and non-B subtypes and within non-B subtypes [10,13,14]. Because of pooling of non-B subtypes in primary data analysis, which limited sample size and power of these studies, the subtype differences escaped detection. Finally, due to the prohibitive costs of tests, detection of resistance mutations was also ignored. Since 2008, we have systematically reviewed evidence on various aspects of non-B subtypes: genetic diversity, drug resistance, prevention of mother to child transmission, and finally, disease progression [15–18].

In this review, we explore the evidence in ART-experienced populations on clinical outcomes (i.e. mortality, CD4 and viral load (VL) changes over time, resistance mutations, death and AIDS).

## Methods

We synthesized global evidence in adults with non-B subtypes for the period January 1996 to January 2013. We searched 11 electronic databases and conference archives (i.e. PubMed, Web-of-Science, Embase, BIOSIS, AIDSLINE, Ovid, PsycINFO, Cochrane Central Register of Controlled Trials, DARE, COCHRANE and ILLUMINA). Search was limited to English language and in humans. References were searched and authors contacted for original data.

Our search string was Search #1: “HIV”[MeSH] OR “HIV-1” [MeSH] OR “HIV-1”[TI]Search #2: “non-b”[TIAB] OR “subtype*”[TI] OR “clade”[TI] OR “strain*”[TI] OR “variant*”[TI] OR “non-B subtype*”[TIAB].

**Figure 1:**
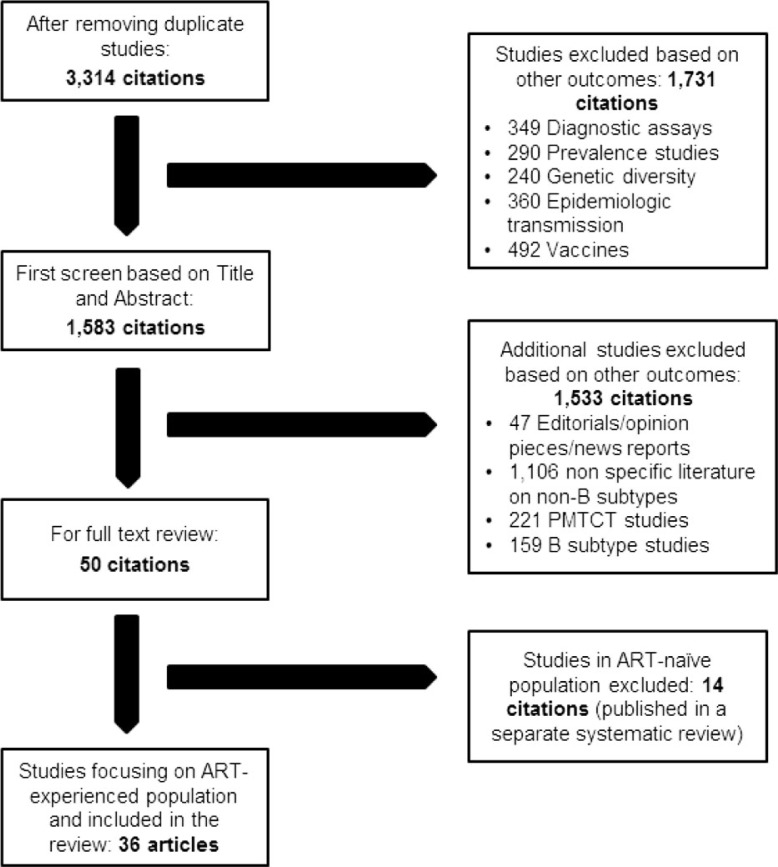
Flow diagram of study inclusion.

Our study selection methodology and exclusion criteria have been elucidated in Figure 1. Studies reporting clinical outcomes such as time to development of AIDS/death, opportunistic infections (OIs), resistance mutations as well as changes in CD4 cell counts and VL were included. Studies on genetic and biochemical diversity, prevention of mother to child transmission or in ART-naïve populations, reviews, editorials, perspectives and news reports were excluded.

Of 10,054 citations retrieved, about 3314 potentially relevant citations were carefully reviewed. A final set of 36 full-text articles and abstracts were selected for this systematic review (Figure 1). Two reviewers (MB, SS) independently searched and abstracted data. One reviewer (MB) abstracted all the data. Two senior reviewers (NPP and JMC) were contacted to resolve disagreements and seek clarifications. A prepiloted data abstraction form was used for abstraction. Variables such as study author, site, country, year, patient population, sample size, subtypes and outcomes were abstracted.

Lack of consistent standardized reporting of measures and outcomes, and use of different baseline comparators, precluded a meta-analysis.

## Results

Overall, considerable heterogeneity in patient populations, countries, sample sizes, and durations of follow-up and comparison groups was noted. Cohort study design formed the majority (30 of 36, 83%) with varying sample sizes (13 to 5268) and follow-up durations (12 to 168 months). Fifty three per cent of studies (19/36) were from Africa (i.e. South Africa, Uganda and Botswana).

Clinical outcomes (i.e. changes in CD4 cell count namely, pre-/post-treatment counts, absolute CD4 and rate of decline, CD4 cell counts at treatment failure) were reported by 40% of the studies. Likewise, changes in VL, time to undetectable VL, pre-/post-treatment VL, VL at treatment failure and viral rebound were reported by 63% studies. Variability in reported thresholds of un-detectability was also noted (i.e. 400 copies/ml vs. 50 copies/ml). Resistance mutations were reported by 53% (19/36). Data on hard outcomes such as mortality and associated morbidities (OIs) were very limited (2/36).

Studies were classified into five groups based on outcomes and non-B subtype comparisons. Refer Supplementary file.

**A) Studies with detailed information on resistance mutations (Table 1)**

We stratified studies on resistance mutations by ART regimen and non-B subtypes. Of 19 studies, 16 (84%) reported specific mutations to nucleoside *reverse transcriptase* inhibitors (NRTIs), non-nucleoside *reverse transcriptase* inhibitors (NNRTIs) and protease inhibitors (PIs), and their proportions in patients on ART (Table 1).

A majority of studies were from developing settings. ART drugs studied were nevirapine (NVP), lamivudine (3TC), zidovudine (AZT), nelfinavir (NFV), abacavir (ABC), efavirenz (EFV), stavudine (d4T), didanosine (ddI), zalcitabine (ddC), ritonavir (RTV), indinavir (IDV), saquinavir (SQV), lopinavir (LPV) and atazanavir (ATV).

Resistance mutations varied across subtypes: for C, F and AE subtypes, the most frequently reported were M184V/I (27 to 78%), K103N (18.6 to 55%) and Y181C (9.3 to 60%) mutations, respectively, in the *reverse-transcriptase* gene. Y181C mutation was present in more than half of the CRF01_AE and CRF02_AG variants in NVP users. L100I mutation after NVP/EFV exposure was present in C (8%) and F (6%) subtypes. High rates of the K65R mutations to d4T were reported in C (9%), CRF01_AE (13%) subtypes [19,20].

A study from Hong Kong [21] found that frequencies of L74V/I and K103N mutations in the *reverse transcriptase* region were different between CRF01_AE (54% and 25%) and subtype B viruses (20% and 50%). Secondary mutation frequencies reported were different for L63P (B>CRF01_AE; *p=*0.043), V77I (exclusively in B; *p*<0.001) and M36I (CRF01_AE>B; *p*<0.001). A study from Brazil [22] found higher frequency of PI-related mutations in codon 63 in subtype B and codon 36 in subtype F. Likewise, PI- (B: 26%; C: 8% and *p=*0.02) and NRTI-related mutations (B: 54%; C: 23% and *p=*0.0012) were significantly different between B and F [23]. Another study from Brazil [24] found that the PI and NNRTI mutations varied across subtypes (PI_ B: 55%; F: 57% and C: 39% and NNRTI_B: 90%; F: 91% and C: 80%). NNRTI mutations were less common in subtype F (B: 64%; F: 54% and C: 62%).

**B) Studies comparing clinical outcomes in individual non-B subtype with B as baseline comparator (Table 2)**

Overall, rates of virologic rebound and suppression varied in A, D and C vs. B, while difference on CD4 declines was observed only in subtype D vs. B. Of 36 studies, only 6 (17%) studies compared clinical outcomes in non-B subtypes with B as baseline comparator.

In a study from Sweden [25], no difference in the rate of CD4 cell decline, clinical progression or plasma VL were reported in subtypes A, B, C or D over a mean follow-up of 44 months (*p*<0.20). In another study, VL suppression was more rapid in subtype C (HR=1.16; 95% CI: 1.01–1.33) and A vs. (HR=1.35; 95% CI: 1.04–1.74) [26]. Rapid virologic rebound was observed in subtype C vs. B (HR=1.40; 95% CI: 1.0–1.95).

In a study from the United Kingdom, a faster rate of virologic rebound for subtype D (70%, *p=*0.02), A (35%, *p=*0.004) and C (34%, *p=*0.01) was observed. Subtype D also showed a four-fold faster CD4 decline relative to subtype B [27]. Two studies from Brazil [24,28] found no difference between subtypes F and B with respect to the number of patients with undetectable VL [28] or virologic failure [24].

**C) Studies comparing clinical outcomes in pooled non-B vs. B subtypes (Table 3)**

Five studies in this subgroup pooled non-B subtypes and used subtype B as baseline comparator to report differences in CD4 cell count and VL counts. In a case-control study from Canada [29], baseline CD4 cell counts were significantly lower in non-B subtypes (*p=*0.02), and the proportion of patients achieving virologic success (VL<400 copies/ml) was 70% in both groups receiving HAART (*p=*0.60). In another study from Belgium, CD4 increase at 24 months was lower in non-B subtypes (161 vs. 235 cells/ml; *p=*0.02) [30]. Although a French study found no significant difference in the time to undetectable VL between non-B (147 days; 95% CI: 119–165) and B subtypes (168 days; 95% CI: 105–234) [31], an Italian study [32] reported a mean CD4 gain of 70 and 156 cells/ml and a mean decrease of VL in B and non-B subtypes of −0.45; −0.79 log copies/ml. The Swiss Cohort study [33] observed a low rate of virologic failure in non-B subtypes (1.4 failures/100 person-years; 95% CI: 0.9–2.1) vs. B (2.6 failures/100 person-years; 95% CI: 2.3–3.0).

**D) Studies in non-B subtypes only (Table 4)**

Of 36 studies, 13 (36%), reported findings on surrogate markers in the non-B subtype prevalent in their setting. Most studies reported findings from single non-B subtypes without comparing it with other subtypes; only one study from Uganda [34] compared two non-B subtypes (A and D), which reported a higher rate of CD4 apoptosis in subtype D vs. A (*p=*0.03). Three studies from Thailand and Cambodia described clinical outcomes for subtype CRF01_AE [20,35,36]. The CD4 cell count at the time of treatment failure was 169 cell/mm^3^ [36]. In the other studies, virologic failure were reported in 64% of patients, and immunologic failure (decrease >30% of the maximum values) was reported in 31% of patients [35].Three studies from South Africa [19,37,38] and one from Botswana [39] reported on clinical outcomes for subtype C. An RCT from South Africa reported survival rate and OIs over 26 months. About 32 deaths (4.9%) and 106 OIs were noted with 271 days as median time to virologic failure [19].

**E) Studies comparing clinical outcomes using ethnicity as a proxy for subtype (Table 5)**

In this subgroup, studies assumed that ethnicity and HIV subtype were correlated [25,40–43]. More specifically, they assumed non-B subtypes were prevalent in Africans and B subtype in Caucasians. Differences were observed for VL and CD4 changes in a few studies. In Spain, significantly higher CD4 gains were observed [43] in Africans compared to Caucasians (*p=*0.027) at 12 months on treatment, but with a loss of difference at 24 months. In a UK study [42], no differences in CD4 cell counts were observed (*p=*0.11), with VL in Africans emerged statistically significantly lower than Caucasians at nine months (*p=*0.001). In Sweden [25], a mean rate of CD4 decline of 2.5 cells/mm^3^/month was found in Africans compared to 2.3 cells/mm^3^/month in matched Swedish controls (*p=*0.87) over a two year period. In another Swedish study [40], 91% of the Caucasians reported an undetectable VL after six months of treatment vs. 77% of Africans (*p=*0.03). Finally, in the United Kingdom [41], the median time to AIDS was nine months (range: 0 to 102 months) in Africans vs.19 months (range: 0 to 156 months) in non-Africans (incidence rate ratio: 1.42; 95% CI: 1.12–1.66). Tuberculosis was more common in Africans (27% vs. 5%) while *Pneumocystis carinii* pneumonia in Caucasians (34% vs.17%). Africans reported a greater risk of AIDS in the crude analysis (HR=1.21; 95% CI: 1.02–1.45) that disappeared in adjusted analyses (adjusted HR=1.15; 95% CI: 0.93–1.43).

## Discussion

We set out to explore the differences in disease progression among the ART-experienced populations with different HIV-1 subtypes with a primary focus on clinical outcomes. Our findings suggest that subtype-specific differences in disease progression do not persist once started on therapy. Furthermore, the use of subtype B-based drug resistance mutation definitions to detect and characterize non-B subtype resistance mutations failed to detect some resistance mutations in non-B subtypes. And although we found corroborating evidence to suggest that the K65R mutation was more frequently selected by d4T in subtype C, it suggested that it might also emerge in CRF_AG at a higher rate than in subtype B [19,44,45]. It is important to highlight that the K65R mutation leads to broad NRTI cross-resistance but hyper-susceptibility to AZT. The prevalence of K65R mutation is increasing due to wider use of therapy with NRTI backbones without AZT [46]. Besides, excessive use of d4T in subtype C and perhaps CRF02_AG also induces K65R in HIV-1. This may complicate achieving virologic control with currently available regimens in resource-poor countries. Thus, the use of d4T should be discouraged in favour of alternative NRTIs. Finally, non-B subtype specific assays (i.e. C, A, D) and preferably point-of-care resistance assays will be needed in order to allow clinicians in resource constrained settings to prescribe the best regimens in the developing world.

Minor findings of our review were: studies in subgroups that compared individuals with non-B vs. B subtypes (Table-2) reported minor differences in clinical outcomes, except those that pooled non-B in one big group obliterating individual level differences. Although data on outcomes such as undetectable VL levels were similar for subtypes B and F, data on death as an outcome was more frequent in subtype B [24,28]. Likewise, CD4 declines and virologic failures were faster in subtype D vs. (C, A and CRF02_AG) [27]. This finding corroborates findings from three other studies that found D to be more aggressive than A [16,34,47].

In the subgroup where non-B subtypes from African settings were pooled, two important differences were reported. CD4 increase at 24 months was lower for non-B subtypes [30] but mean durations of follow-up in these studies were shorter [31], suggesting a poorer treatment response. This finding could indicate an immune-deficient status with low CD4 levels at ART initiation. However, these differences could also be due to social or ethnic characteristics, if African immigrants that belong to a lower socio-economic strata who were perhaps not able to continue with ART.

In contrast, in the Swiss cohort study where African immigrants with non-B subtypes were studied, a low virologic failure rate in non-B subtypes was reported [33]. This contrasting finding was ascribed to the following factors: 1) variable CD4 and VL thresholds used for initiating HAART, and 2) a better nutritional status of immigrant patients in developed settings.

In other studies, it was impossible to elucidate patterns from reports where disease progression was described in individual subtypes (such as subtypes C and AE) without any baseline comparators. Barring one study that compared subtypes A and D, a majority reported no comparators [34]. This could be an oversight on the part of investigators or lack of subtypes in the setting where studies were conducted. Regardless, they failed to provide meaningful information.

However, in studies, where ethnicity was used as a surrogate for subtypes, one study found that time to develop AIDS was faster in Africans as compared to Caucasians, whereas the percentage of patients achieving undetectable VL was higher in Caucasians than Africans [41]. In contrast, some other studies found no differences, and concluded that ethnicity could confound the relationship between HIV subtype and disease progression. A study from the United Kingdom reported unadjusted estimates with a higher risk of progression to AIDS for Africans vs. non-Africans that were rendered non-significant with adjustment for ethnicity in multivariate models [41]. The conflicting results of these analyses are due to the fact that, in most studies, ethnicity and HIV-1 subtypes are related in a way that resists statistical separation: B subtypes affect mainly Caucasian in industrialized settings and non-Bs affect African and other ethnicities in non-industrialized settings. Hence, in studies available to date, the HIV-1 subtype as a variable is a surrogate for potentially confounding variables (i.e. ethnicity, socioeconomic status and other comorbidities) that could significantly affect progression of HIV-1 infection further. It is impossible to study and delineate these issues in the context of observational studies. The only way to study them is in the context of randomized controlled trials, which are harder to conduct because many settings do not have prevalent B’s and non-B subtypes and more so treatment naive participants that could be followed over time to document these outcomes.

In the same vein, resistance tests are done on patients in Africa after a long time, compared to the Western countries. NNRTI-based regimens are known to induce NRTI resistance and that too quite rapidly, whereas PI’s with a high genetic barrier protect against development of NRTI resistance. However, most non-B subtypes are treated by NNRTI’s and this poses a threat towards development of resistance and biases inferences of studies that fail to take this effect of regimens into account. Regardless, this concern is of paramount importance for the future sustainability of ART regimens in resource poor settings.

As alluded to before, in two other studies, a lack of access to health care, food insecurity, variable exposure to environmental pathogens, and variable levels of immune-suppression were deduced to be the most likely causes of reduced survival with AIDS, rather than subtype [25,41]. These have emerged in recent years to affect clinical disease progression in developing settings.

Finally, even though minor differences in changes in CD4 cell count existed between ethnicities, other factors such as socioeconomic status, cultural differences influencing behaviour that affected treatment adherence, and others mentioned above played a bigger role in affecting clinical outcomes over time [48]. As is known in literature, other factors such as differences in access to health care and poorer engagement with the health care network can also profoundly influence clinical disease progression [42,48]. In sum, these factors are essential in strategic planning initiatives that optimize effectiveness of ART.

### Implications for future research

Differences observed between HIV subtypes prior to initiation of ART disappear with its initiation and with patients on treatment [15]. However, given the lack of consistent data in this field, research in well-designed studies, with adequate sample sizes, powered to detect differences between non-B subtypes (i.e. C, A, D, AE, AG) vs. subtypes (preferably, non-B, or a consistent comparator B) are needed. Perhaps, larger databases that store information on non-B subtypes could be an invaluable resource for making such direct comparisons [49]. Finally, studying patterns in non-B subtypes (by themselves, without comparators) also adds to the pool of data and provide directions on developing targeted treatment strategies.

### Implications for practice

There is need to record HIV genotypes at baseline both in practice and in research, more so in global settings. To do so, it is necessary to develop and introduce simple point-of-care resistance assays. These assays should be capable of detecting highly deleterious resistance mutations (e.g. K65R) and that information will guide better treatment decisions. Lastly, standardization of treatment guidelines has been a long awaited need. Thus, the recently released WHO HIV treatment guidelines are greatly welcomed. These guidelines have now recommended the combination of tenofovir plus emtricitabine or laminvudine as first-line NRTI backbone [50]. This recommendation, if followed, should result in a shift that will further minimize the impact of HIV genetic diversity on drug resistance and disease progression across most non-B subtypes. Failure to do so in the long term will minimize the global benefit of ART.

### Strengths and limitations

To our knowledge, this is the first systematic review on clinical outcomes in treated HIV-1 non-B subtypes. The methodology of this review was rigorous and was performed independently by two reviewers. Limitations include the following: 1) heterogeneity in prevalence of subtypes, with respect to the baseline comparators, 2) outcomes examined, 3) variable reporting of measures of effect (risk ratios, hazard ratios, odds ratios) and use of variable cut-offs for virologic failure that precluded pooling in a meta-analysis, 4) reporting of null findings (possible publication bias) and 5) small sample size in some studies with shorter durations of follow up that added no new knowledge.

## Conclusions

Genetic diversity amongst non-B subtypes was minimized with initiation of ART but variability in resistance mutations across non-B subtypes was observed. A lack of reporting of baseline genotypic data was attributed to limited access to resistance assays in developing settings, and that limited inference. With newer and refined WHO ART guidelines there will be a need to carefully survey resistance to HIV in areas dominated by non-B subtypes. To fill that need, POC resistance assays will emerge as a highly relevant area of applied research.
